# Bis(acetohydroxamato-κ^2^ *O*,*O*′)diphenyl­tin(IV)

**DOI:** 10.1107/S160053681102558X

**Published:** 2011-07-06

**Authors:** Caihong Yue, Qingkun Wu, Handong Yin

**Affiliations:** a College of Chemistry and Chemical Engineering, Liaocheng University, Shandong 252059, People’s Republic of China

## Abstract

The complex mol­ecule of the title compound, [Sn(C_6_H_5_)_2_(C_2_H_4_NO_2_)_2_], has crystallographically imposed twofold symmetry. The Sn atom is coordinated by four O atoms from two acetohydroxamate ligands and by two C atoms from phenyl groups in a distorted octa­hedral geometry. In the crystal, mol­ecules are connected by N—H⋯O hydrogen-bonding inter­actions, forming a chain structure along the *c* axis

## Related literature

For the biological activity of diorganotin(IV) complexes with hydroxamates, see: Shang *et al.* (2007 ▶). For a related structure, see: Harrison *et al.* (1976 ▶). For van der Waals radii, see: Bondi (1964 ▶).
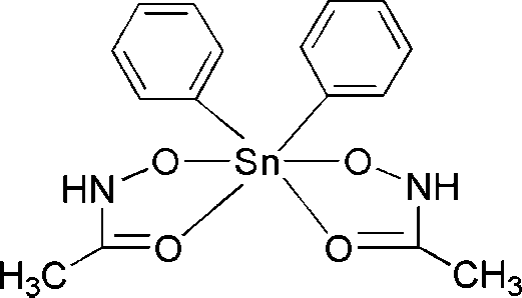


## Experimental

### 

#### Crystal data

[Sn(C_6_H_5_)_2_(C_2_H_4_NO_2_)_2_]
*M* *_r_* = 421.01
Monoclinic, 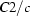

*a* = 18.7713 (17) Å
*b* = 10.2683 (8) Å
*c* = 9.8326 (6) Å
β = 112.842 (1)°
*V* = 1746.6 (2) Å^3^
*Z* = 4
Mo *K*α radiation
μ = 1.48 mm^−1^
*T* = 298 K
0.38 × 0.33 × 0.19 mm

#### Data collection

Siemens SMART CCD area-detector diffractometer
Absorption correction: multi-scan (*SADABS*; Sheldrick, 1996 ▶) *T*_min_ = 0.603, *T*_max_ = 0.766
4295 measured reflections
1542 independent reflections
1367 reflections with *I* > 2σ(*I*)
*R*_int_ = 0.048

#### Refinement

*R*[*F* ^2^ > 2σ(*F* ^2^)] = 0.030
*wR*(*F* ^2^) = 0.077
*S* = 1.00
1542 reflections
106 parameters
H-atom parameters constrained
Δρ_max_ = 0.72 e Å^−3^
Δρ_min_ = −0.94 e Å^−3^

### 

Data collection: *SMART* (Siemens, 1996 ▶); cell refinement: *SAINT* (Siemens, 1996 ▶); data reduction: *SAINT*; program(s) used to solve structure: *SHELXS97* (Sheldrick, 2008 ▶); program(s) used to refine structure: *SHELXL97* (Sheldrick, 2008 ▶); molecular graphics: *SHELXTL* (Sheldrick, 2008 ▶); software used to prepare material for publication: *SHELXTL*.

## Supplementary Material

Crystal structure: contains datablock(s) I, global. DOI: 10.1107/S160053681102558X/rz2612sup1.cif

Structure factors: contains datablock(s) I. DOI: 10.1107/S160053681102558X/rz2612Isup2.hkl

Additional supplementary materials:  crystallographic information; 3D view; checkCIF report

## Figures and Tables

**Table 1:** Hydrogen-bond geometry (Å, °)

*D*—H⋯*A*	*D*—H	H⋯*A*	*D*⋯*A*	*D*—H⋯*A*
N1—H1⋯O1^i^	0.86	2.03	2.847 (4)	159

Symmetry code: (i) 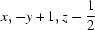.

**Fig. 1.**
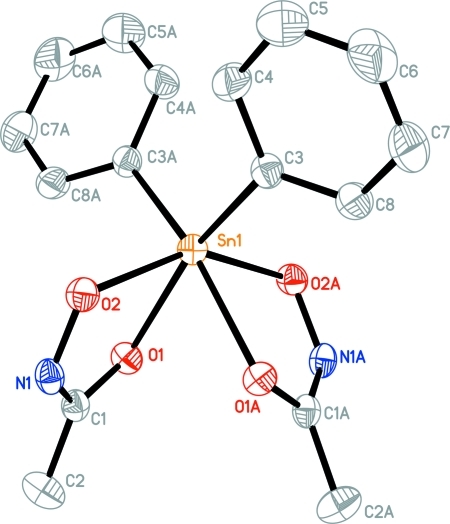
The molecular structure of the compound, showing 50% probability displacement ellipsoids. H atoms are omitted for clarity. Symmetry code: (A) = -x, y, -z + 1/2.

**Fig. 2.**
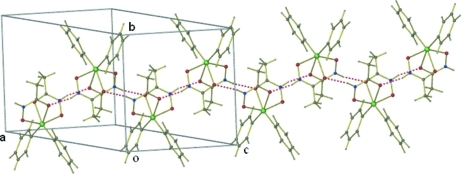
View of the one-dimensional linear chain structure in the title compound.

## supplementary crystallographic information

### Comment

Among many multidentate organic ligands, hydroxamic acids are of particular importance, because of their remarkable structural diversity and biological applications (Shang *et al.* , 2007). As a continuation of our interest in this area, we have synthesized the title compound and report its crystal structure herein.

The molecular structure of the compound is depicted in Fig. 1. The complex molecule has crystallographically imposed two-fold symmetry. The tin atom is six-coordinated in a distorted octahedral geometry. The Sn–O bond distances (Sn1–O2 = 2.103 (2) Å; Sn1–O = 2.275 (3) Å) are close to the sum of the covalent radii (2.13 Å; Bondi, 1964), and the Sn–C distance (Sn1–C3 = 2.147 (4) Å) are in the range observed in a related compound (2.14–2.18 Å; Harrison *et al.*, 1976). In the crystal packing, the molecules are linked by N—H···O hydrogen bonds (Table 1) into a one-dimensional chains parallel to the *c* axis (Fig. 2).

### Experimental

The reaction was carried out under nitrogen atmosphere. Acetohydroxamic acid (0.4 mmol) and KOH (0.4 mmol) in methanol (30 ml) were added to a Schlenk flask and stirred for 30 min. Diphenyltin dichloride (0.2 mmol) was then added to the reactor. The reaction mixture was stirred for 8 h at room temperature and then filtrated. The filtrate was evaporated *in vacuo* to dryness. The obtained solid was recrystallized from a dichloromethane-petroleum ether (3:1 *v*/*v*) solution (yield 86%; m. p. 431 K). Anal. Calcd (%) for C_16_H_18_N_2_O_4_Sn (Mr = 421.01): C, 45.64; H, 4.31; N, 6.65; O, 15.20. Found (%): C, 45.60; H, 4.25; N, 6.62; O,15.16.

### Refinement

All H atoms were positioned with idealized geometry (C—H = 0.83-0.96 Å; N—H = 0.86 Å) and were refined isotropically with *U*_iso_(H) = 1.2 *U*_eq_(C, N) or 1.5 *U*_eq_(C) for methyl H atoms.

### Crystal data

**Table:**

[Sn(C_6_H_5_)_2_(C_2_H_4_NO_2_)_2_]	*F*(000) = 840
*M**_r_* = 421.01	*D*_x_ = 1.601 Mg m^−^^3^
Monoclinic, *C*2/*c*	Mo *K*α radiation, λ = 0.71073 Å
Hall symbol: -C 2yc	Cell parameters from 2602 reflections
*a* = 18.7713 (17) Å	θ = 2.3–26.2°
*b* = 10.2683 (8) Å	µ = 1.48 mm^−^^1^
*c* = 9.8326 (6) Å	*T* = 298 K
β = 112.842 (1)°	Block, colourless
*V* = 1746.6 (2) Å^3^	0.38 × 0.33 × 0.19 mm
*Z* = 4	

### Data collection

**Table:**

Siemens SMART CCD area-detector diffractometer	1542 independent reflections
Radiation source: fine-focus sealed tube	1367 reflections with *I* > 2σ(*I*)
graphite	*R*_int_ = 0.048
phi and ω scans	θ_max_ = 25.0°, θ_min_ = 2.3°
Absorption correction: multi-scan (*SADABS*; Sheldrick, 1996)	*h* = −22→14
*T*_min_ = 0.603, *T*_max_ = 0.766	*k* = −12→11
4295 measured reflections	*l* = −11→11

### Refinement

**Table:**

Refinement on *F*^2^	Primary atom site location: structure-invariant direct methods
Least-squares matrix: full	Secondary atom site location: difference Fourier map
*R*[*F*^2^ > 2σ(*F*^2^)] = 0.030	Hydrogen site location: inferred from neighbouring sites
*wR*(*F*^2^) = 0.077	H-atom parameters constrained
*S* = 1.00	*w* = 1/[σ^2^(*F*_o_^2^) + (0.0322*P*)^2^ + 6.1631*P*] where *P* = (*F*_o_^2^ + 2*F*_c_^2^)/3
1542 reflections	(Δ/σ)_max_ < 0.001
106 parameters	Δρ_max_ = 0.72 e Å^−^^3^
0 restraints	Δρ_min_ = −0.94 e Å^−^^3^

### Fractional atomic coordinates and isotropic or equivalent isotropic displacement parameters (Å^2^)

**Table:**

	*x*	*y*	*z*	*U*_iso_*/*U*_eq_	
Sn1	0.0000	0.27983 (4)	0.2500	0.03426 (15)	
O1	−0.07784 (14)	0.4550 (3)	0.2287 (3)	0.0377 (6)	
O2	−0.04374 (15)	0.3345 (3)	0.0264 (3)	0.0399 (6)	
N1	−0.07653 (16)	0.4552 (3)	0.0036 (3)	0.0371 (7)	
H1	−0.0874	0.4929	−0.0802	0.045*	
C1	−0.0919 (2)	0.5148 (4)	0.1067 (4)	0.0356 (8)	
C2	−0.1242 (3)	0.6484 (5)	0.0813 (5)	0.0599 (12)	
H2A	−0.1762	0.6467	0.0766	0.090*	
H2B	−0.1241	0.6812	−0.0101	0.090*	
H2C	−0.0932	0.7039	0.1608	0.090*	
C3	0.0918 (2)	0.1575 (4)	0.2454 (4)	0.0393 (9)	
C4	0.0752 (3)	0.0355 (5)	0.1751 (5)	0.0591 (12)	
H4	0.0244	0.0061	0.1339	0.071*	
C5	0.1343 (4)	−0.0411 (6)	0.1669 (7)	0.0833 (19)	
H5	0.1228	−0.1216	0.1200	0.100*	
C6	0.2094 (3)	0.0010 (7)	0.2273 (7)	0.0827 (18)	
H6	0.2485	−0.0494	0.2182	0.099*	
C7	0.2269 (3)	0.1177 (6)	0.3013 (6)	0.0710 (15)	
H7	0.2781	0.1450	0.3452	0.085*	
C8	0.1685 (2)	0.1946 (5)	0.3103 (5)	0.0506 (11)	
H8	0.1811	0.2731	0.3612	0.061*	

### Atomic displacement parameters (Å^2^)

**Table:**

	*U*^11^	*U*^22^	*U*^33^	*U*^12^	*U*^13^	*U*^23^
Sn1	0.0343 (2)	0.0369 (2)	0.0340 (2)	0.000	0.01590 (15)	0.000
O1	0.0429 (14)	0.0422 (16)	0.0321 (13)	0.0038 (12)	0.0190 (11)	0.0018 (11)
O2	0.0462 (15)	0.0414 (16)	0.0324 (13)	−0.0006 (13)	0.0158 (11)	−0.0028 (12)
N1	0.0322 (16)	0.049 (2)	0.0299 (16)	−0.0002 (14)	0.0117 (13)	0.0062 (14)
C1	0.0300 (18)	0.043 (2)	0.0332 (19)	−0.0028 (16)	0.0122 (15)	0.0030 (16)
C2	0.073 (3)	0.055 (3)	0.061 (3)	0.019 (3)	0.036 (2)	0.015 (2)
C3	0.040 (2)	0.043 (2)	0.0341 (19)	0.0034 (18)	0.0137 (16)	−0.0027 (17)
C4	0.057 (3)	0.051 (3)	0.061 (3)	0.003 (2)	0.014 (2)	−0.017 (2)
C5	0.089 (4)	0.069 (4)	0.087 (4)	0.020 (3)	0.029 (3)	−0.031 (3)
C6	0.068 (4)	0.091 (5)	0.091 (4)	0.033 (3)	0.034 (3)	−0.012 (4)
C7	0.043 (3)	0.080 (4)	0.087 (4)	0.014 (3)	0.022 (2)	0.002 (3)
C8	0.042 (2)	0.046 (3)	0.063 (3)	0.0045 (19)	0.019 (2)	−0.003 (2)

### Geometric parameters (Å, °)

**Table:**

Sn1—O2^i^	2.103 (2)	C2—H2C	0.9600
Sn1—O2	2.103 (2)	C3—C8	1.383 (6)
Sn1—C3^i^	2.147 (4)	C3—C4	1.406 (6)
Sn1—C3	2.147 (4)	C4—C5	1.388 (7)
Sn1—O1^i^	2.275 (3)	C4—H4	0.9300
Sn1—O1	2.275 (3)	C5—C6	1.371 (9)
O1—C1	1.281 (4)	C5—H5	0.9300
O2—N1	1.363 (4)	C6—C7	1.374 (8)
N1—C1	1.308 (5)	C6—H6	0.9300
N1—H1	0.8600	C7—C8	1.381 (7)
C1—C2	1.482 (6)	C7—H7	0.9300
C2—H2A	0.9600	C8—H8	0.9300
C2—H2B	0.9600		
			
O2^i^—Sn1—O2	149.02 (16)	C1—C2—H2B	109.5
O2^i^—Sn1—C3^i^	97.18 (12)	H2A—C2—H2B	109.5
O2—Sn1—C3^i^	100.81 (12)	C1—C2—H2C	109.5
O2^i^—Sn1—C3	100.81 (12)	H2A—C2—H2C	109.5
O2—Sn1—C3	97.18 (12)	H2B—C2—H2C	109.5
C3^i^—Sn1—C3	108.4 (2)	C8—C3—C4	117.7 (4)
O2^i^—Sn1—O1^i^	73.53 (10)	C8—C3—Sn1	122.0 (3)
O2—Sn1—O1^i^	82.02 (10)	C4—C3—Sn1	120.3 (3)
C3^i^—Sn1—O1^i^	162.29 (13)	C5—C4—C3	120.2 (5)
C3—Sn1—O1^i^	88.41 (13)	C5—C4—H4	119.9
O2^i^—Sn1—O1	82.02 (10)	C3—C4—H4	119.9
O2—Sn1—O1	73.53 (10)	C6—C5—C4	120.5 (5)
C3^i^—Sn1—O1	88.41 (13)	C6—C5—H5	119.7
C3—Sn1—O1	162.29 (13)	C4—C5—H5	119.7
O1^i^—Sn1—O1	75.54 (13)	C5—C6—C7	119.9 (5)
C1—O1—Sn1	110.9 (2)	C5—C6—H6	120.1
N1—O2—Sn1	112.5 (2)	C7—C6—H6	120.1
C1—N1—O2	121.3 (3)	C6—C7—C8	120.0 (5)
C1—N1—H1	119.4	C6—C7—H7	120.0
O2—N1—H1	119.4	C8—C7—H7	120.0
O1—C1—N1	118.3 (4)	C7—C8—C3	121.5 (5)
O1—C1—C2	121.6 (4)	C7—C8—H8	119.2
N1—C1—C2	120.1 (3)	C3—C8—H8	119.2
C1—C2—H2A	109.5		
			
O2^i^—Sn1—O1—C1	144.7 (2)	C3^i^—Sn1—C3—C8	−148.3 (4)
O2—Sn1—O1—C1	−16.0 (2)	O1^i^—Sn1—C3—C8	26.0 (4)
C3^i^—Sn1—O1—C1	−117.8 (3)	O1—Sn1—C3—C8	50.8 (6)
C3—Sn1—O1—C1	44.1 (5)	O2^i^—Sn1—C3—C4	133.1 (4)
O1^i^—Sn1—O1—C1	69.8 (2)	O2—Sn1—C3—C4	−72.2 (4)
O2^i^—Sn1—O2—N1	−24.99 (19)	C3^i^—Sn1—C3—C4	31.7 (3)
C3^i^—Sn1—O2—N1	99.4 (2)	O1^i^—Sn1—C3—C4	−154.0 (4)
C3—Sn1—O2—N1	−150.2 (2)	O1—Sn1—C3—C4	−129.2 (4)
O1^i^—Sn1—O2—N1	−62.9 (2)	C8—C3—C4—C5	−2.7 (7)
O1—Sn1—O2—N1	14.3 (2)	Sn1—C3—C4—C5	177.3 (4)
Sn1—O2—N1—C1	−12.8 (4)	C3—C4—C5—C6	0.1 (9)
Sn1—O1—C1—N1	15.1 (4)	C4—C5—C6—C7	2.3 (10)
Sn1—O1—C1—C2	−164.6 (3)	C5—C6—C7—C8	−2.2 (10)
O2—N1—C1—O1	−2.5 (5)	C6—C7—C8—C3	−0.4 (9)
O2—N1—C1—C2	177.2 (3)	C4—C3—C8—C7	2.8 (7)
O2^i^—Sn1—C3—C8	−46.9 (4)	Sn1—C3—C8—C7	−177.2 (4)
O2—Sn1—C3—C8	107.8 (4)		

Symmetry codes: (i) −*x*, *y*, −*z*+1/2.

### Hydrogen-bond geometry (Å, °)

**Table:**

*D*—H···*A*	*D*—H	H···*A*	*D*···*A*	*D*—H···*A*
N1—H1···O1^ii^	0.86	2.03	2.847 (4)	159

Symmetry codes: (ii) *x*, −*y*+1, *z*−1/2.
